# Corneal Endothelial Microscopy: Does a Manual Recognition of the Endothelial Cells Help the Morphometric Analysis Compared to a Fully Automatic Approach?

**DOI:** 10.3390/vision8040064

**Published:** 2024-10-30

**Authors:** Giulia Carlotta Rizzo, Rosa Di Grassi, Erika Ponzini, Silvia Tavazzi, Fabrizio Zeri

**Affiliations:** 1 Department of Materials Sciences, University of Milano-Bicocca, 20125 Milan, Italy; digrassir@gmail.com (R.D.G.); erika.ponzini@unimib.it (E.P.); silvia.tavazzi@unimib.it (S.T.); fabrizio.zeri@unimib.it (F.Z.); 2 Research Centre in Optics and Optometry (COMiB), University of Milano-Bicocca, 20125 Milan, Italy; 3 School of Life and Health Sciences, Aston University, Birmingham B4 7ET, UK

**Keywords:** corneal endothelium, specular microscopy, endothelium morphology, endothelial cell density, coefficient of variation, hexagonality

## Abstract

This study investigated whether manual integration in the recognition of the endothelial cells produces different outcomes of morphometric parameters compared to a fully automatic approach. Eight hundred and ninety endothelial images, originally acquired by the Perseus Specular Microscope (CSO, Florence, Italy), from seven positions of right and left corneas were selected from the database of the Research Centre in Optics and Optometry at the University of Milano-Bicocca. For each image selected, two procedures of cell identification were performed by the Perseus: an automatic identification and a manual-integrated procedure to add potential additional cells with the available editing tool. At the end of both procedures, the endothelial cell density (ECD), coefficient of variation (CV), and hexagonality (HEX) of the mosaic were calculated. The HEX in the two procedures was significantly different for all comparisons (*p* < 0.001), but clinically negligible. No significant differences were found for the CV and ECD in the images of both eyes irrespective of the corneal position of acquisition (except for ECD in three corneal portions, *p* < 0.05). To conclude, it is possible to recognise a significantly higher number of cells using the manual-integrated procedure than it is using the fully automatic one, but this does not change the morphological parameters achieved.

## 1. Introduction

The assessment of corneal endothelium morphology is clinically relevant since its structural anomalies can be connected to alterations in the physiology of the cornea. In particular, the level of corneal hydration, which is responsible for corneal transparency, is mainly controlled by the proper functioning of this layer [1,2]. For example, the endothelium assessment is extremely important in cataract patients before surgery since the possible decrease in the number of corneal endothelial cells induced by the surgical procedure might lead to corneal oedema in patients with a low endothelial cell density (ECD) [3,4,5,6,7].

Corneal endothelial assessment is also of primary importance for the diagnosis and treatment of many ocular conditions such as Fuchs endothelial corneal dystrophy, posterior polymorphous corneal dystrophy, congenital hereditary endothelial dystrophy, pseudoexfoliation keratopathy, and keratoconus, or in monitoring the impact of anterior chamber inflammation on the cornea [8,9,10,11]. Furthermore, endothelial parameters are evaluated to establish the outcomes in terms of safety of other surgical procedures beyond cataract surgery such as refractive surgery, corneal collagen crosslinking, Descemet stripping automated endothelial keratoplasty (DSEK), Descemet’s membrane endothelial keratoplasty (DMEK), corneal graft, and glaucoma surgery [12,13,14,15,16,17,18,19,20,21]. Changes in endothelial parameters are also investigated in response to topical drugs [22,23].

Endothelium monitoring can also be a powerful tool for contact lens wearers, since hypoxia conditions induced by contact lens wear have been linked to an increase in the variability of cell size (polymegathism) and shape (pleomorphism) [24,25,26] or in the monitoring of corneal structural changes after orthokeratology [27]. Finally, endothelium assessment is relevant to monitor corneal endothelium health during ageing [28].

The main parameters that characterise the corneal endothelium, largely reported in research and clinical studies, are the ECD, the hexagonality (HEX), and the coefficient of variation (CV) [1,29,30]: ECD is defined as the number of cells per unit area (cells/mm^2^); HEX, which is the key indicator for pleomorphism, is the percentage of six-sided cells; and CV is the ratio between the standard deviation of the mean cell area and the mean cell area that expresses the level of polymegathism.

In clinical practice, an in vivo morphometric assessment and characterisation of the endothelium can be carried out by specular microscopy [3,31,32,33], which can be performed either with a slit lamp or with stand-alone devices such as contact and non-contact specular microscopes, allowing for the observation of the corneal endothelium using the principle of specular reflection [34,35]. This technique has been utilised since Vogt’s original description over a century ago [32,36]. The introduction of stand-alone devices for quantitative analysis, initially by Maurice in 1968 [37], allowed the examination of enucleated eyes, and, soon after, these were adapted for both contact and non-contact use in patients [31,34]. Over the past four decades, non-contact specular microscopes have gained popularity in clinical settings due to their non-invasive nature—eliminating the need for anaesthesia and minimizing the risks of corneal trauma or cross-infection. These microscopes, which include models like the Konan Noncon Robo SP8000, CC7000, CellChek XL, Topcon SP-1000, SP 2000P Image-NET, SP3000P, Tomey EM-3000, CSO Perseus, and NIDEK CEM-530, feature automatic image focusing technology that requires minimal practical skills, provides a wide field of view, and offers automated cell detection along with computer-assisted calculation of key morphological parameters. Studies have been conducted on these models to assess measurement reliability and interchangeability [38,39,40,41,42,43,44,45]. A critical aspect of these devices is their ability to identify cell boundaries automatically, which forms the basis for morphometric analysis. While these devices offer manual editing of cell boundary identification, increasing the number of analysable cells, the criteria for boundary identification may differ between manual and automatic systems. Historically, some fully automated systems have been deemed unreliable, with semi-automated or manual methods, though more time-consuming, providing greater accuracy [46,47]. Furthermore, analysing the endothelium in the presence of guttae is even more complex, as the software may not recognise guttae as abnormal areas devoid of normal endothelial cells, necessitating manual identification [48,49] or the application of recently developed artificial intelligence-based approaches [50].

The purpose of this work was to evaluate the agreement between the morphological parameters achieved after an automatic process of morphological evaluation of the cell boundaries and after a manual-integrated procedure to add potential additional cells, both performed by the specular microscope Perseus (CSO, Florence, Italy).

## 2. Materials and Methods

This retrospective masked crossover study was approved by the Board of Optics and Optometry of the host University (doc. n° 4/2019, May 2019).

### 2.1. Instrumentation

The instrument used to carry out the study was the non-contact specular microscope Perseus (CSO, Florence, Italy). This device acquires endothelium images by a guided three-dimensional automatic acquisition [51] and an optical magnification of 180×. Seven images are acquired from different positions of the cornea. After a first measure in the centre (central; C), the fixation point shifts eccentrically to allow acquisition in a peripheral position in the opposite portion of the cornea: inferior nasal (IN), inferior central (I), inferior temporal (IT), superior temporal (ST), superior central (S), and superior nasal (SN). Once the seven images have been acquired, the instrument software automatically identifies a certain number of cells by the corner method to analyse the endothelial cells that compound the endothelial mosaic for each image [43,51]. An index of reliability of the quality of acquisition is calculated as the percentage of the area where cells are identified (on which the morphometric analysis will be performed) divided by the total area of the acquired image (0.15 mm^2^) [43,51]. The index is then reported using a three-level colour scale: green for values greater than 50%, yellow for values between 30% and 50%, and red for values below 30% (unable to process data). A series of morphometric parameters of the endothelial image are also calculated, including ECD, HEX, and CV.

The instrument software offers an editing tool that allows the operator to manually detect cell boundaries that have not been recognised or modify boundaries that have been wrongly detected by the automatic method. The operator can draw a line on the boundary or delete the boundary with a pen and delete tools, respectively. After manual editing, the morphometric parameters are recalculated by the same algorithms applied in the fully automatic procedure. In some cases, guttae are present in the images. In this case, the editing tool can be used to identify the boundaries of the guttae. After this, the software also provides a functional ECD (fECD), expressed in cells/mm^2^, which represents the actual cell density calculated excluding the area occupied by the guttae.

### 2.2. Procedure

**Figure 1:**
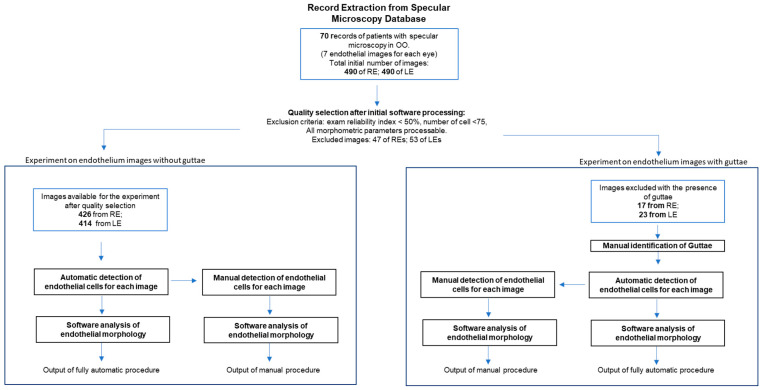
Flow diagram of the study design. OO: both eyes. RE: right eye. LE: left eye.

The study design is resumed in Figure 1. Seventy records of seventy patients (mean age 55 ± 10 years; range 30–79 years; fourteen females) who underwent specular microscopy by Perseus (CSO, Florence, Italy) for both eyes were extracted from the database of the Research Centre in Optics and Optometry of the University of Milano-Bicocca (COMiB), anonymising any personal information. Each record contained, for each eye, seven endothelial images, one for each of the seven different corneal positions described in the Section 2.1, namely C, IN, I, IT, ST, S, and SN. Therefore, 490 images were initially available both for the right eyes (REs) and the left eyes (LEs) of the entire sample. A quality assessment of each image was performed by employing the following exclusion criteria:

▪ An index of reliability provided by the instrument between 30% and 50% (see previous paragraph);
▪ A number of cells automatically identified by the software lower than 75 units [52];
▪ Images in which the algorithm was not able to calculate the morphological parameters taken into consideration in this study (see previous paragraph).

Fifty-six cases were excluded due to a low index of reliability: twenty-four for the right eye (RE) and thirty-two for the left eye (LE). Nine cases were excluded due to the low number of cells automatically identified: five for the RE and four for the LE. Thirty-five cases were excluded due to the absence of calculated morphological parameters: eighteen for the RE and seventeen for the LE.

The images that passed the quality assessment were divided into two sub-sets:

▪ *Images of endothelium without guttae* (426 RE and 414 LE);
▪ *Images of endothelium with guttae* (17 RE and 23 LE).

**Figure 2:**
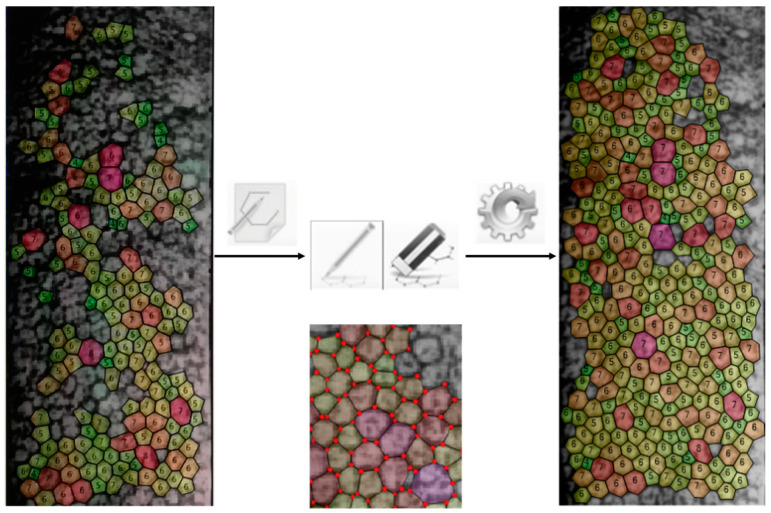
Example of the manual editing of the image provided by the instrument.

In the first sub-set (*images of endothelium without guttae*), two procedures were performed to achieve the parameters of the endothelial mosaic by the software of the Perseus specular microscope:

▪ *Fully automatic procedure*: After an automatic procedure to identify cell boundaries, four morphometric parameters of the endothelial mosaic–number of cells identified, ECD, HEX, and CV– were calculated.
▪ *Manual procedure*: Images processed by the automatic procedure to identify cell boundaries were provided to an operator who was previously trained in the use of the instrument software tool to edit cell boundaries. This operator was a pre-registration optometrist who received extensive training on the instrument before starting the study, with over 80 endothelial images manually edited under the supervision of a lecturer with over 25 years of experience in endothelial microscopy. This training was considered suitable to reach a good level of reliability, considering that the operator’s sole task was to add and correct the identification of endothelial cells and to report the reprocessed data. The decision to use a single, well-trained operator for the manual editing phase was made to avoid introducing bias that could arise from the involvement of multiple operators. This operator, masked to the final output of the fully automatic procedure, was assigned the task of performing a manual-integrated procedure with the specific instruction to adjust the incorrectly shaped boundaries, eliminate incorrectly recognised cells, or add cells that were not recognised at all (Figure 2). After the manual editing, the four morphometric parameters of the endothelial mosaic were recalculated.

**Figure 3:**
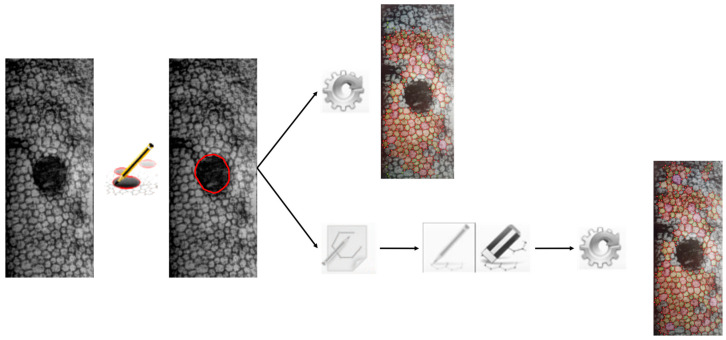
Example of the manual guttae detection and automatic image processing, with (below) and without manual editing of the image provided by the instrument.

In the second sub-set (images of endothelium with guttae), manual identification of guttae boundaries was first required since guttae are not automatically recognised by the instrument software (Figure 3). After guttae selection, the same two procedures, fully automatic and manual, used for the first sub-set were performed and the four morphometric parameters were calculated (Figure 1). In this case, functional ECD (fECD), which is the cell density in the area excluding the area covered by guttae, was considered rather than ECD, i.e., the number of cells/mm^2^ calculated on the total processed area (<0.15 mm^2^). The total examination area was always 0.15 mm^2^.

### 2.3. Statistical Analysis

For the sub-set of images *without guttae* (426 and 414 for RE and LE, respectively), the analysis was carried out separately both for the eyes and the seven corneal positions. This was done to avoid a possible bias linked to the fact that measurements obtained from the right and left eye of a subject are often correlated [53].

Conversely, for the sub-set of images *with guttae*, the analysis was carried out clustered in one single group of images taken from two eyes and different corneal positions due to the reduced number of images available (N = 40; 17 from RE and 23 from LE).

The normality of the distributions was assessed by the Shapiro–Wilk test. Comparisons between the two procedures were carried out using a paired t-test if both distributions (*fully automatic* and *manual*) were normally distributed or a Wilcoxon test if at least one of the two distributions was not normally distributed. Correlation between the two procedures was investigated by calculating the Pearson or Spearman Rho correlation coefficients for normally distributed and not normally distributed data, respectively. Bland–Altman plots were used to assess the difference in measurement between the two procedures as a function of the mean of the two measurements [54]. This kind of plot enables the detection of any systematic trend in the differences between the fully automatic procedure and the manual procedure due to an increase in the amplitude of the outcome examined. The presence of a proportional bias in the Bland–Altman plot was explored by examining the correlation between the average and the difference between the two measurements. The statistical analysis was performed using SPSS, version 29 (IBM Corp., Armonk, NY, USA).

## 3. Results

### 3.1. Study on Images Without the Presence of Guttae

Table 1 and Table 2 show the analytic number of images available as a function of the different corneal positions, the main statistical descriptives of the four parameters analysed in the study (the number of cells detected, ECD, HEX, and CV), and the comparisons for each parameter and corneal position between the fully automatic and manual procedures.

The analysis consistently showed, in both eyes and all corneal positions, a higher number of cells detected by the manual procedure compared to the fully automatic procedure. As far as the ECD is concerned, statistical differences between the two procedures have been found only in one out of seven corneal positions and in two out of seven corneal positions for the RE and LE, respectively. No difference between the two procedures was found for CV for both eyes and all corneal positions, whereas the HEX results always showed statistical differences between the two procedures with higher values achieved by the manual procedure ranging from 2% to 4% depending on the corneal position. Finally, correlations between measures achieved with the fully automatic and manual procedures resulted in statistical significance for all four variables in the two eyes.

**Figure 4:**
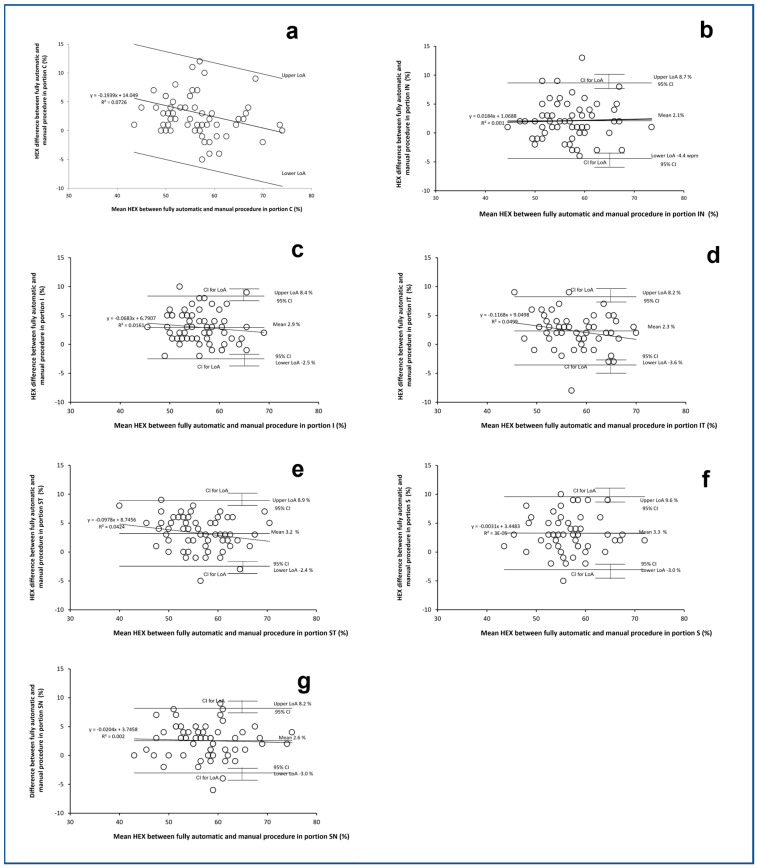
Bland–Altman plots of the differences between HEX (%) achieved by the fully automatic and manual procedures plotted against the mean HEX (%) between the fully automatic and manual procedures. The seven plots refer to the seven different corneal positions captured with specular microscope in the right eyes. (**a**): central (C); (**b**): inferior nasal (IN); (**c**): inferior central (I); (**d**): inferior temporal (IT); (**e**): superior temporal (ST); (**f**): superior central (SC); (**g**): superior nasal (SN). Limits of Agreement (LoA) are calculated as mean difference ± 1.96 SD of differences, Confidence interval (CI) at 95%.

**Figure 5:**
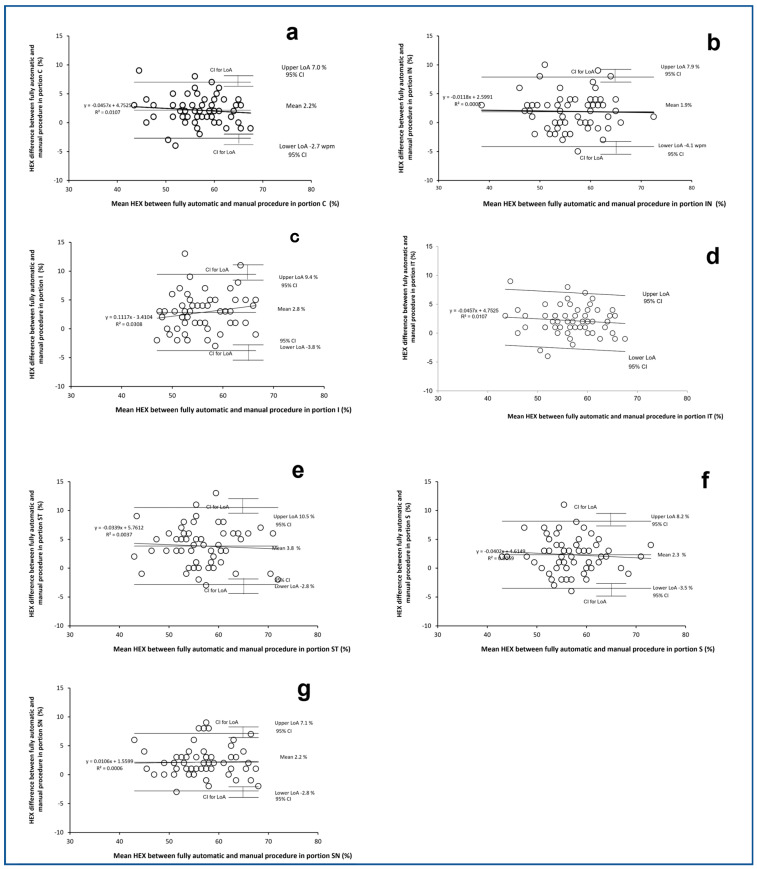
Bland–Altman plots of the differences between HEX (%) achieved by the fully automatic and manual procedures plotted against the mean HEX (%) between the fully automatic and manual procedures. The seven plots refer to the seven different corneal positions captured with specular microscope in the left eyes. (**a**): central (C); (**b**): inferior nasal (IN); (**c**): inferior central (I); (**d**): inferior temporal (IT); (**e**): superior temporal (ST); (**f**): superior central (SC); (**g**): superior nasal (SN). Limits of Agreement (LoA) are calculated as mean difference ± 1.96 SD of differences, Confidence interval (CI) at 95%.

Considering that differences between the fully automatic and manual procedures have been consistently found only for HEX, it has been decided to show Bland–Altman plots only for the results of HEX in Figure 4 and Figure 5 for the right eye and left eye, respectively. In each figure, seven Bland–Altman plots are shown for the seven portions of the cornea: a (C), b (IN), c (l), d (IT), e (ST), f (S), and g (SN). Only in the portion C of the RE (Figure 4a) and in the portion IT of the LE (Figure 4d) does the Bland–Altman plot show a significant correlation (Spearman rho = −0.33; *p* < 0.01 and r of Pearson 0.28; *p* = 0.04, respectively). This indicates that the difference in HEX between the two methods became significantly more positive with the reduction of HEX.

### 3.2. Experiment on Images with the Presence of Guttae

As previously mentioned in the data analysis section, due to the reduced number of images available (N = 40; 17 from the RE and 23 from the LE), all the images presenting guttae were grouped and analysed regardless of the eye and the corneal positions. This choice is also justified by the outcomes from the previous sample of images not presenting guttae, demonstrating that the eye and the corneal positions did not affect the trend in the difference between the fully automatic and manual procedures, which was consistently significant only for the number of cells detected and HEX.

In the second group of images, guttae covered 1.0 ± 1.2% (range 0.5–5.0%) of the total area. Descriptive statistics of endothelial morphometric parameters for this group are reported along with paired comparisons between the procedures in Table 3. In this case, fECD rather than ECD was reported.

**Figure 6:**
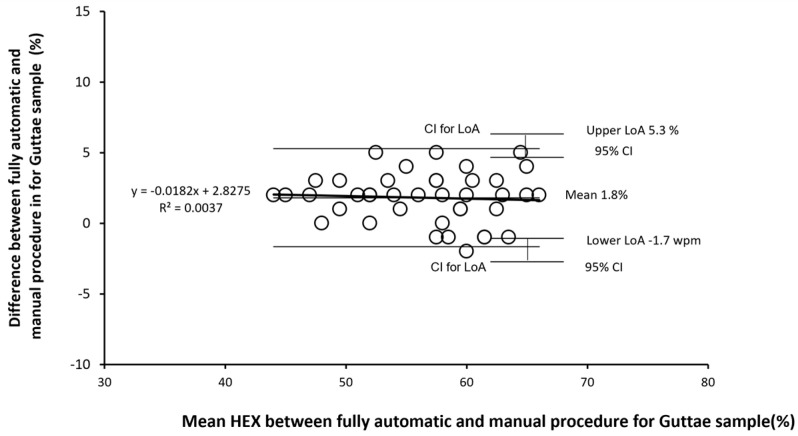
Bland–Altman plot of the differences between HEX (%) achieved by the fully automatic and manual procedures plotted against the mean HEX (%) between the fully automatic and manual procedures on images with the presence of guttae. Limits of Agreement (LoA) are calculated as mean difference ± 1.96 SD of differences, Confidence interval (CI) at 95%.

The scenario in this sub-sample is very similar to that achieved for images not presenting guttae, with statistical differences between the fully automatic and manual procedures limited to the number of cells detected (as expected) and HEX, and correlations between measures achieved with the fully automatic and manual procedures always statistically significant. Also, for the sub-sample of images with guttae, the Bland–Altman plot is shown only for the results of HEX (Figure 6). The Bland–Altman plot does not show any proportional bias.

## 4. Discussion

Nowadays, non-contact specular microscopes have achieved excellent performance in automatic alignment, autofocusing, capturing high-quality endothelial images, the computer-assisted detection of cell boundaries, and calculating morphometric parameters using various algorithms [29,38,39,40,41,42,43,44,45,55]. Nonetheless, some devices, such as the Perseus specular microscope, offer software tools to manually enhance cell boundary detection [43,51]. This study investigated whether manual integration in the recognition of the endothelial cells produces different outcomes of morphometric parameters compared to a fully automatic approach.

The study showed that the manual editing of the image to detect other possible cells not captured by the fully automated procedure was extremely time-consuming. On average, the trained operator needed from 20 min to 30 min to edit, in one image, the cell boundaries missed/wrongly detected by the fully automated procedure. However, the manual editing was almost limited to the addition of cells not detected rather than the correction of wrong boundaries inserted by the automatic procedure, which occurred for a maximum of 2/3 cells for each image. In contrast, the fully automatic procedure completed the task of identifying cell boundaries and calculating endothelial mosaic parameters in a few seconds per image. Given that a trained operator required significantly more time, it can be assumed that, eventually, for an untrained operator, it would take an even longer time, potentially increasing both the time required and the risk of errors.

This manual procedure made it possible to detect a significantly higher number of cells compared to the fully automatic one. In images from the sub-group without guttae, the increase ranged from between 32.4% and 45.0% depending on the eye and the portions of cornea considered (Table 1 and Table 2), the mean (±std dev) of the percentual increase being 39.8% (±3.8%). In the sub-sample of images with guttae, the increase was 36.9% (Table 3). Despite the significant increase in the number of cells detected, this did not determine changes in the outcomes of ECD and CV. Specifically, the manual procedure caused a slight statistically significant increase in ECD in the “SN” corneal portion of the RE and a decrease in the “I” and “ST” corneal portions of the LE, but these changes were negligible: 17, 16, and 74 cells/mm^2^, respectively. This lack of difference represents an advancement compared to earlier studies, where fully automated procedures provided unreliable ECD data compared to manual approaches [46]. Recent studies have shown varying results, although the manual procedures used to trace cell contours or correct false identification of borders cannot be considered perfectly equivalent to those used in the present study. Price et al. [56] demonstrated that the differences in ECD estimates between fully automatic and manual procedures vary with different devices and the types of corneas analysed (normal vs. post-Descemet’s stripping endothelial keratoplasty, DSEK). The Tomey EM 3000 (Tomey, Nagoya, Japan) automated cell detection program produced ECD readings comparable to manual methods both in normal and DSEK corneas. The Konan Noncon Robo SP-8800 (Konan Medical, Hyogo, Japan) automated program provided similar results in normal corneas but differed in DSEK images. The Nidek Confoscan 4 (Nidek Co., Ltd., Gamamori, Japan) showed different ECD results between the automated and manual procedures in both normal and DSEK corneas. Scarpa and Ruggeri [57] found no differences in ECD and other endothelial parameters between procedures in both normal and pathological corneas. Regarding CV, a previous study demonstrated that a semi-automated image analysis system (Topcon IMAGEnet) and a manual method led to similar results [58]. In contrast, this study found that HEX was consistently higher after the manual procedure than the fully automated procedure for both eyes and all corneal positions, although the increase was clinically negligible, ranging from 2 to 5%. Similar results were found in the sub-sample of images with guttae. This result agrees with the findings of Cheung and Cho [47]. One possible explanation for the increase in HEX after the manual editing is that the operator might introduce a bias in detecting six-sided cells because this shape corresponds to the observer’s expectations.

A potential limitation of this study is that the initial sample consisted of subjects with normal corneas, and the images from all the corneal regions were of high quality. For example, the number of cells automatically detected was ≥75 units, which is above the threshold needed for reliable parameter estimation [52]. It is known that automated methods may struggle to accurately determine endothelial parameters from poor-quality images [44]. This study did not include a sample of poor quality images, and even the sub-sample of images with guttae included only slightly degraded images, with a maximum of 5.0% of the total area covered by guttae. Another limitation is that only a single operator conducted the study, so no data about inter-operator variability is available. Additionally, in the subgroup of specular images with guttae, the approach of excluding areas covered by guttae from calculations is acknowledged as a limitation. This method can lead to an overestimation of cell density, as has been shown in previous research [50].

## 5. Conclusions

The study showed that manual editing by a trained operator to detect additional possible cells detectable in images acquired by the non-contact specular microscope Perseus (CSO, Florence, Italy) did not change, in a clinically significant way, the estimation of the main endothelial morphological parameters studied (ECD, CV, and HEX) compared to a fully automatic analysis performed by the instrument. This was achieved for images of corneas of good quality either in the absence of guttae or in a sub-sample of images where guttae covered a maximum of 5.0% of the total area examined. These results do not suggest the use of the extremely time-consuming manual procedure when the quality of the image achieved by the instrument is sufficient to allow fully automatic processing, i.e., with a number of cells detected higher than 75.

## Figures and Tables

**Table 1:** Descriptive statistics of endothelial morphometric parameters (number of cells, endothelial cell density—ECD, coefficient of variation—CV, and hexagonality HEX) for the RE achieved from the fully automatic and manual procedures as a function of the different corneal positions. Paired comparisons between the procedure and correlations are also shown. Statistical tests utilised were the paired *t*-test for paired comparisons and the Pearson coefficient for correlation if both distributions (fully automatic and manual) were normally distributed. The Wilcoxon test and Spearman Rho correlation coefficient were used if at least one of the two distributions (fully automatic and manual) was not normally distributed.

Right Eye												
	Fully Automatic Procedure				Manual Procedure				Comparison and Correlation Between the Two Procedures			
Corneal Position (N. Images Available)	N. of Cells Mean ± SD (Range)	ECD (Cells/mm^2^) Mean ± SD (Range)	CV (%) Mean ± SD (Range)	HEX (%) Mean ± SD (Range)	N. of Cells Mean ± SD (Range)	ECD (Cells/mm^2^) Mean ± SD (Range)	CV (%) Mean ± SD (Range)	HEX (%) Mean ± SD (Range)	N. of Cells	ECD	CV	HEX
C (63)	213 ± 37 (130–287)	2487 ± 266 (1874–3004)	34 ± 5 (24–45)	55 ± 8 (33–74)	293 ± 37 (207–353)	2507 ± 272 (1869–3065)	34 ± 6 (24–48)	58 ± 6 (44–74)	**t = −20.0, *p* < 0.001;** **r = 0.64, *p* < 0.001**	t = −1.5, *p* = 0.14; **r = 0.92, *p* < 0.001**	t = −0.1, *p* = 0.95; **r = 0.94, *p* < 0.001**	**t = −5.2, *p* < 0.001;** **r = 0.78, *p* < 0.001**
IN (61)	209 ± 35 (168–303)	2579 ± 274 (1907–3096)	34 ± 5 (26–45)	56 ± 6 (44–73)	294 ± 36 (198–352)	2584 ± 266 (1911–3082)	34 ± 5 (26–45)	58 ± 6 (45–74)	**t = −20.2, *p* < 0.001;** **r = 0.57, *p* < 0.001**	t = −0.5, *p* = 0.60; **r = 0.96, *p* < 0.001**	Wilcoxon = 0.7, *p* = 0.52; **Spearman Rho = 0.93, *p* < 0.001**	**t = −4.9, *p* < 0.001;** **r = 0.84, *p* < 0.001**
I (61)	189 ± 39 (98–292)	2492 ± 311 (1153–2972)	34 ± 5 (24–45)	55 ± 5 (44–68)	271 ± 44 (125–361)	2498 ± 328 (1169–3041)	34 ± 4 (25–44)	58 ± 5 (47–70)	**Wilcoxon** **= 6.8, *p* < 0.001;** **Spearman Rho = 0.50, *p* < 0.001**	Wilcoxon = 0.7, *p* = 0.47; **Spearman Rho = 0.97, *p* < 0.001**	Wilcoxon = −0.5, *p* = 0.61; **Spearman Rho = 0.87, *p* < 0.001**	**t = −4.9, *p* < 0.001;** **r = 0.87, *p* < 0.001**
IT (56)	205 ± 41 (111–299)	2517 ± 313 (1277–3223)	34 ± 5 (26–47)	56 ± 6 (41–69)	282 ± 45 (151–366)	2532 ± 327 (1279–3176)	34 ± 5 (25–48)	59 ± 6 (48–71)	**t = −19.9, *p* < 0.001;** **r = 0.77, *p* < 0.001**	Wilcoxon = 0.7, *p* = 0.50; **Spearman Rho = 0.95, *p* < 0.001**	Wilcoxon = −0.9, *p* = 0.35; **Spearman Rho = 0.87, *p* < 0.001**	**t = −5.8, *p* < 0.001;** **r = 0.88, *p* < 0.001**
ST (62)	199 ± 41 (115–294)	2632 ± 314 (1626–3154)	35 ± 5 (27–47)	55 ± 7 (36–68)	280 ± 55 (119–359)	2636 ± 326 (1585–3171)	35 ± 5 (27–49)	58 ± 6 (44–73)	**Wilcoxon** **= 6.9; *p* < 0.001** **Spearman Rho = 0.77, *p* < 0.001**	t = −0.6, *p* = 0.52; **r = 0.99, *p* < 0.001**	Wilcoxon = 1.0; *p* = 0.31; **Spearman Rho = 0.96, *p* < 0.001**	**t = −8.8, *p* < 0.001;** **r = 0.90, *p* < 0.001**
S (59)	186 ± 45 (109–290)	2664 ± 316 (2013–3270)	36 ± 5 (27–49)	55 ± 6 (43–71)	268 ± 50 (157–370)	2676 ± 320 (1931–3286)	36 ± 5 (28–46)	58 ± 6 (44–73)	**t = −19.1, *p* < 0.001;** **r = 0.76, *p* < 0.001**	t = −1.4, *p* = 0.17; **r = 0.98, *p* < 0.001**	Wilcoxon = −0.6, *p* = 0.54; **Spearman Rho = 0.90, *p* < 0.001**	**t = −7.8, *p* < 0.001;** **r = 0.83, *p* < 0.001**
SN (64)	206 ± 39 (125–332)	2676 ± 302 (1925–3301)	35 ± 5 (27–49)	56 ± 7 (43–73)	291 ± 46 (130–387)	2693± 303 (1898–3307)	35 ± 5 (26–49)	59 ± 6 (43–77)	**Wilcoxon** **= 7.0;** ***p* < 0.001;** **Spearman Rho = 0.58, *p* < 0.001**	**t = −2.2, *p* = 0.04;** **r = 0.98, *p* < 0.001**	Wilcoxon = 0.2; *p* = 0.88; **Spearman Rho = 0.96, *p* < 0.001**	**t = −7.2, *p* < 0.001;** **r = 0.90, *p* < 0.001**

(C: central; IN: inferior nasal; I: inferior central; IT: inferior temporal; ST: superior temporal; S: superior central; SN: superior nasal).

**Table 2:** Descriptive statistics of endothelial morphometric parameters (number of cells, endothelial cell density—ECD, coefficient of variation—CV, and hexagonality HEX) for the LE achieved from the fully automatic and manual procedures as a function of the different corneal positions. Paired comparisons between the procedure and correlations are also shown. Statistical tests utilised were the paired *t*-test for comparison and the Pearson coefficient for correlation if both distributions (fully automatic and manual) were normally distributed. The Wilcoxon test and Spearman correlation were used if at least one of the two distributions (fully automatic and manual) was not normally distributed.

Left Eye												
	Fully Automatic Procedure				Manual Procedure				Comparison and Correlation Between the Two Procedures			
Corneal Position (N. Images Available)	N. of Cells Mean ± SD (Range)	ECD (Cells/mm^2^) Mean ± SD (Range)	CV (%) Mean ± SD (Range)	HEX (%) Mean ± SD (Range)	N. of Cells Mean ± SD (Range)	ECD (Cells/mm^2^) Mean ± SD (Range)	CV (%) Mean ± SD (Range)	HEX (%) Mean ± SD (Range)	N. of Cells	ECD	CV	HEX
C (59)	216 ± 39 (122–310)	2497 ± 264 (1803–2962)	34 ± 5 (25–46)	56 ± 6 (40–68)	286 ± 40 (177–360)	2497 ± 269 (1816–3006)	34 ± 5 (26–46)	58 ± 6 (45–67)	**t = −21.0; *p* < 0.001** **r = 0.79, *p* < 0.001**	t = 0.1; *p* = 0.96 **r = 0.98, *p* < 0.001**	t = −0.2; *p* = 0.81 **r = 0.94, *p* < 0.001**	**t = −6.7; *p* < 0.001** **r = 0.91, *p* < 0.001**
IN (63)	207 ± 48 (100–309)	2597 ± 278 (1818–3108)	34 ± 5 (24–48)	56 ± 6 (37–72)	284 ± 51 (124–386)	2606 ± 289 (1822–3215)	34 ± 5 (26–47)	58 ± 6 (40–73)	**Wilcoxon** **= 6.9;** ***p* < 0.001** **Spearman Rho = 0.70, *p* < 0.001**	t = −1.2; *p* = 0.23 **r = 0.98, *p* < 0.001**	Wilcoxon = 0.1; *p* = 0.92 **Spearman Rho = 0.94, *p* < 0.001**	**t = −4.9; *p* < 0.001** **r = 0.88, *p* < 0.001**
I (54)	185 ± 38 (106–277)	2535 ± 262 (1865–3065)	34 ± 5 (26–50)	55 ± 5 (46–67)	266 ± 47 (132–363)	2519 ± 259 (1860–3024)	34 ± 5 (26–49)	57 ± 6 (46–69)	**t = −18.7; *p* < 0.001** **r = 0.74, *p* < 0.001**	**t = 2.4; *p* = 0.02** **r = 0.98, *p* < 0.001**	Wilcoxon = −1.1; *p* = 0.28 **Spearman Rho = 0.93, *p* < 0.001**	**t = −6.2; *p* < 0.001** **r = 0.82, *p* < 0.001**
IT (54)	189 ± 42 (100–281)	2508 ± 328 (1098–3066)	34 ± 5 (27–45)	55 ± 6 (42–68)	274 ± 51 (130–372)	2520 ± 332 (1093–3062)	35 ± 5 (27–46)	57 ± 5 (46–69)	**Wilcoxon** **= 6.4;** ***p* < 0.001** **Spearman Rho = 0.67, *p* < 0.001**	Wilcoxon = 0.8; *p* = 0.44 **Spearman Rho = 0.95, *p* < 0.001**	Wilcoxon = 1.5; *p* = 0.13 **Spearman Rho = 0.90, *p* < 0.001**	**t = −5.5; *p* < 0.001** **r = 0.83, *p* < 0.001**
ST (60)	200 ± 40 (101–303)	2594 ± 296 (1878–3157)	35 ± 5 (27–48)	55 ± 6 (39–73)	279 ± 49 (105–363)	2520 ± 332 (1093–3062)	35 ± 5 (33–50)	59 ± 6 (44–74)	**Wilcoxon** **= 6.7;** ***p* < 0.001** **Spearman Rho = 0.62, *p* < 0.001**	**t = −2.7; *p* = 0.01** **r = 0.98, *p* < 0.001**	Wilcoxon = −0.3; *p* = 0.78 **Spearman Rho = 0.95, *p* < 0.001**	**t = −8.7; *p* < 0.001** **r = 0.86, *p* < 0.001**
S (61)	197 ± 45 (114–323)	2689 ± 302 (1924–3226)	35 ± 5 (25–47)	56 ± 6 (42–71)	277 ± 54 (130–381)	2691 ± 293 (1919–3222)	35 ± 5 (26–50)	58 ± 6 (31–75)	**Wilcoxon** **= 6.7;** ***p* < 0.001** **Spearman Rho = 0.77, *p* < 0.001**	t = −0.4; *p* = 0.35 **r = 0.98, *p* < 0.001**	Wilcoxon = 0.8; *p* = 0.40 **Spearman Rho = 0.90, *p* < 0.001**	**t = −6.1; *p* < 0.001** **r = 0.87, *p* < 0.001**
SN (63)	222 ± 39 (122–294)	2692 ± 275 (1930–3201)	35 ± 6 (25–50)	55 ± 6 (40–69)	297 ± 47 (176–373)	2702 ± 289 (1926–3300)	35 ± 6 (26–52)	57 ± 6 (46–70)	**Wilcoxon** **= 6.9;** ***p* < 0.001 Spearman Rho = 0.70, *p* < 0.001**	t = −1.5; *p* = 0.14 **r = 0.98, *p* < 0.001**	Wilcoxon = 0.3; *p* = 0.81 **Spearman Rho = 0.95, *p* < 0.001**	**t = −6.7; *p* < 0.001** **r = 0.91, *p* < 0.001**

(C: central; IN: inferior nasal; I: inferior central; IT: inferior temporal; ST: superior temporal; S: superior central; SN: superior nasal).

**Table 3:** Descriptive statistics of endothelial morphometric parameters (number of cells, endothelial cell density—ECD, coefficient of variation—CV, and hexagonality HEX) for the group of images presenting guttae achieved from the fully automatic and manual procedures. Paired comparisons between the procedure and correlations are also shown. The statistical tests utilised were the paired *t*-test for comparison and the Pearson coefficient for correlation if both distributions (fully automatic and manual) were normally distributed. The Wilcoxon test and Spearman correlation were used if at least one of the two distributions (fully automatic and manual) was not normally distributed.

	Fully Automatic Procedure				Manual Procedure				Comparison Between the Two Procedures			
N. Images Available	N. of Cells Mean ± SD (Range)	ECD (cells/mm^2^) Mean ± SD (Range)	CV (%) Mean ± SD (Range)	HEX (%) Mean ± SD (Range)	N. of Cells Mean ± SD (Range)	ECD (cells/mm^2^) Mean ± SD (Range)	CV (%) Mean ± SD (Range)	HEX (%) Mean ± SD (Range)	N. of Cells	ECD	CV	HEX
40	198 ± 38 (123–265)	2501 ± 327 (1400–3068)	33 ± 4 (27–42)	56 ± 6 (43–65)	271 ± 57 (126–346)	2516 ± 334 (1400–3179)	33 ± 4 (28–42)	57 ± 6 (45–67)	**Wilcoxon** **= 5.5;** ***p* < 0.001** **Spearman Rho = 0.78, *p* < 0.001**	Wilcoxon = 1.4; *p* = 0.16 **Spearman Rho = 0.97, *p* < 0.001**	Wilcoxon = −0.02; *p* = 0.99 **Spearman Rho = 0.89, *p* < 0.001**	**t = −6.4; *p* < 0.001** **r = 0.96, *p* < 0.001**

## Data Availability

The datasets generated during and/or analysed during the current study are available from the corresponding author on reasonable request.
